# Network Modularity in Breast Cancer Molecular Subtypes

**DOI:** 10.3389/fphys.2017.00915

**Published:** 2017-11-17

**Authors:** Sergio Antonio Alcalá-Corona, Guillermo de Anda-Jáuregui, Jesús Espinal-Enríquez, Enrique Hernández-Lemus

**Affiliations:** ^1^Computational Genomics, National Institute of Genomic Medicine, México City, Mexico; ^2^Centro de Ciencias de la Complejidad, Universidad Nacional Autónoma de México, México City, Mexico; ^3^School of Medicine and Health Sciences, University of North Dakota, Grand Forks, ND, United States

**Keywords:** network modularity, gene regulatory networks (GRN), breast cancer subtypes, pathway enrichment analysis, Functional modules, community structure

## Abstract

Breast cancer is a heterogeneous and complex disease, a clear manifestation of this is its classification into different molecular subtypes. On the other hand, gene transcriptional networks may exhibit different modular structures that can be related to known biological processes. Thus, modular structures in transcriptional networks may be seen as manifestations of regulatory structures that tightly controls biological processes. In this work, we identify modular structures on gene transcriptional networks previously inferred from microarray data of molecular subtypes of breast cancer: luminal A, luminal B, basal, and HER2-enriched. We analyzed the modules (communities) found in each network to identify particular biological functions (described in the Gene Ontology database) associated to them. We further explored these modules and their associated functions to identify common and unique features that could allow a better level of description of breast cancer, particularly in the basal-like subtype, the most aggressive and poor prognosis manifestation. Our findings related to the immune system and a decrease in cell death-related processes in basal subtype could help to understand it and design strategies for its treatment.

## Introduction

### Breast cancer: a heterogeneous disease

Breast cancer is the malignant neoplasy with the highest incidence and mortality among women worldwide (Ferlay et al., 2014). One of the main challenges for its treatment is its heterogeneous nature, with manifestations spanning over a multitude of clinical, physiological and survival variants, resulting in differences in available therapeutic options (Polyak, 2011).

The heterogeneity of breast cancer can be traced down to the genetic level. Molecular subtyping provides a helpful tool to classify tumors by identifying common patterns in their genetic expression. Several classification algorithms that allow the classification of breast samples into *molecular subtypes* using different technological platforms have been developed (Perou et al., 2000; Guedj et al., 2012). A common classification scheme is given in terms of four main molecular subtypes which are luminal A, luminal B, HER2-enriched and basal-like.

#### Luminal A

Around a half of the total cases of breast cancer correspond to luminal A tumors (Fan et al., 2006). These tumors are often positive to estrogen receptor (*ER*) and negative to *ERBB2* receptor, they also present overexpression of the *ER*-regulated genes. This subtype usually has the best prognosis (Hu et al., 2006) and the lower recurrence rates (Arvold et al., 2011; Metzger-Filho et al., 2013).

#### Luminal B

Despite the fact that this subtype has a similar expression pattern to that of luminal A, it is characterized by a higher variability in the *ER* expression and a higher expression of proliferative genes, also mutations associated with *TP53* and genetic instability have been found in it. Around 20% of the total of breast cancer tumors corresponds with this phenotype (Sørlie et al., 2001) which tends to have poorer prognosis than luminal A tumors (Haque et al., 2012).

#### HER2-enriched

This intrinsic subtype is characterized by the overexpression of the *ERBB2* receptor, which is associated with chromosomal-level amplification (Burstein, 2005). These tumors are negative for estrogen and progesterone receptors and have a poorer prognosis than those of luminal subtypes (Yang et al., 2011).

#### Basal

20% of breast tumors are basal-like and the majority of *triple negative* belong to this subtype. Unlike the subtypes described above, basal-like tumors have underexpression of the estrogen, progesterone and *ERBB2* receptors. These tumors are also associated with higher genetic instability, they are more aggressive and present the poorest prognosis. The majority of *BRCA*-1 mutations-related tumors belong to this subtype (Voduc et al., 2010; Haque et al., 2012; Bayraktar and Glück, 2013; Metzger-Filho et al., 2013; Singha et al., 2016) and have a gene expression profile similar to the *basal mammary epithelium*.

Basal subtype cannot be treated by conventional hormone therapy or monoclonal antibodies (Perou et al., 2000). Usually, the treatment for patients with this subtype includes surgery, radiation therapy and chemotherapy. This is one important reason to study the genome-wide level connectivity patterns of genes associated with the appearance of basal subtype tumors.

Taking into account that basal tumors do not present known targets to targeted therapy, searching for functional modules that may be targeted results appealing. By using molecular signatures that functionally map to specific processes, development of directed therapies that provide alternatives to the canonical ones may become reachable. In this sense, the use of novel technologies may grant insights to reach a better understanding of the differences and similarities between distinct manifestations of breast cancer, and perhaps design new strategies against it (Espinal-Enríquez et al., 2017a).

Phenotypic variations among breast cancer subtypes arise due to differences in their underlying regulatory programs (de Anda-Jáuregui et al., 2016). Said differences in the global structure of subtype-specific gene regulatory networks may indeed reflect differences in lower scales of regulation, in particular in the presence of underlying functional modules, as we will discuss further in this work.

### Modularity in transcriptional networks

The amount of genomic data available nowadays is huge and the biological information hidden in it is valuable. Using novel theoretical frameworks to analyze and understand the complex mechanisms underlying the relationships between molecules becomes a must. This is the case of the gene regulatory network approach, which uses gene expression data to model and describe the relationship between thousands of genes under particular phenotypes. In this theoretical representation, nodes (also called vertices) represent genes, while the links are an instance of interaction or relationship between those genes. These models are usually given as complex networks (Albert and Barabási, 2002; Dorogovtsev and Mendes, 2002; Newman, 2003, 2010; Boccaletti et al., 2006; Caldarelli and Vespignani, 2007).

Global organization patterns of large complex networks involve the presence of structural sub-units (subnetworks) that have been called *communities* or *modules*, broadly defined as subsets of tightly interconnected nodes so that the density of *within-connections* is higher than that of *between-connections* (Girvan and Newman, 2002; Porter et al., 2009; Fortunato, 2010). Module detection in networks, is still an open problem in computer science (Mucha et al., 2010) and there is an important variety of methods and algorithms to detect such communities (Gulbahce and Lehmann, 2008; Ahn et al., 2010; Fortunato, 2010; Xie et al., 2013; Fortunato and Hric, 2016); thus, modular structure is an issue of particular relevance, from economic and social systems (Espinal-Enríquez et al., 2015b; García-Pérez et al., 2016), to biological systems (Alcalá-Corona et al., 2016).

In the case of gene transcriptional regulatory networks (Tang et al., 2012), a module or community may correspond to a co-regulated set of genes (Wilkinson and Huberman, 2004; Zhu et al., 2008; Marbach et al., 2012; Cantini et al., 2015). In this sense, modules topology may capture some aspects of the phenomenology behind biological processes. Previous works have identified module detection as a valuable alternative for the identification of groups of genes that can associate biological features to phenotypes (Cantini et al., 2015; Alcalá-Corona et al., 2016). We followed along the lines of Alcalá-Corona et al. (2016) looking to identify biologically functional modules. We were able to find subtype-specific functional processes in modules detected by using a random-walk based community detection algorithm (Rosvall and Bergstrom, 2008).

Recently, it has been shown how the differences in transcriptional programs between breast cancer molecular subtypes are reflected in their specific transcriptional networks (de Anda-Jáuregui et al., 2016). A remaining question is whether these subtype-specific networks contain modules—i.e., subnetworks—that may be associated to known biological features. Therefore, in this work we explore the modular structure of previously inferred (de Anda-Jáuregui et al., 2016) breast cancer molecular subtype transcriptional networks for: luminal A, luminal B, basal, and HER2-enriched tumors.

For further analyses, we decided to focus in the basal subtype, since it is the one with a poorer prognosis and less options for treatment. For this subtype we identified specific communities statistically enriched for processes related to the immune system. Among these, the community containing the *PSMB9* gene—composed by only 21 genes—is also the only one enriched for apoptosis-related events. Furthermore, the expression signature of molecules associated with the proteasome complex indicates that cell death-related events are strongly decreased. We suggest that communities found with this approach must be further investigated experimentally looking for alternative therapeutic options in the spirit of personalized medicine (Espinal-Enríquez et al., 2017b; Hernández-Lemus et al., 2017).

## Materials and methods

**Figure 1:**
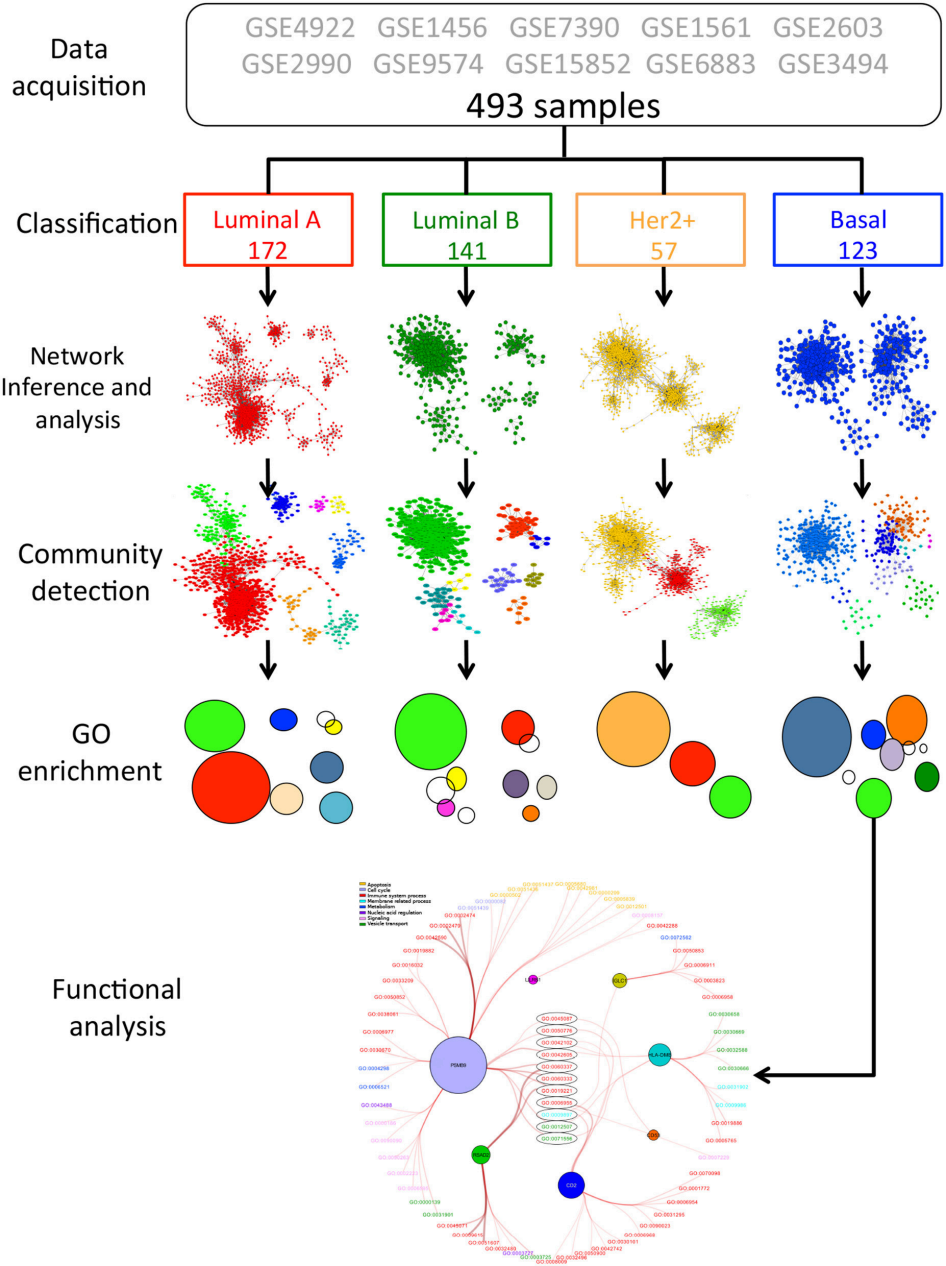
Workflow followed in this study. The pipeline starts with the collection of 493 breast cancer samples. PAM50 classification was performed as in (de Anda-Jáuregui et al., 2015). In the image, the colors correspond to each molecular subtype. For the four subtypes, mutual information-based networks (de Anda-Jáuregui et al., 2016) were inferred with ARACNE (Margolin et al., 2006). Once networks were built, we detected communities for each subtype network by using Infomap (Rosvall and Bergstrom, 2008). Modules are represented by different colored nodes. Detected modules were later enriched using HTSanalyzeR (Wang et al., 2011). In the picture, enriched communities are full colored circles, meanwhile non-enriched ones are non colored. Finally, the enriched modules were analyzed at a more profound level, by observing the most general processes that are involved in these modules. In the picture, enriched communities found in basal subtype are classified according to upper general processes. Each process is represented with a different color and the labels correspond to the GO-ID.

A complete workflow for this study is depicted in Figure 1. This section is divided as follows: data acquisition and classification, network inference, community detection, Gene Ontology enrichment analysis and functional analysis.

### Breast cancer microarray data and classification

For this analysis, we used a set of 493 microarray expression profiles for breast cancer samples processed on the Affymetrix HGU133A platform. This experimental dataset was collected from the Gene Expression Omnibus (GEO) from accession numbers GSE4922 (Ivshina et al., 2006), GSE1456 (Pawitan et al., 2005), GSE7390 (Desmedt et al., 2007), GSE1561 (Farmer et al., 2005), GSE2603 (Minn et al., 2005), GSE2990 (Sotiriou et al., 2006), GSE9574 (Tripathi et al., 2008), GSE15852 (Ni et al., 2010), GSE6883 (Liu et al., 2007), and GSE3494 (Miller et al., 2005). Data preprocessing was performed following a pipeline for *Robust Multi-array Average* (Irizarry et al., 2003), as in Tovar et al. (2015). Breast cancer samples were classified using the well-validated PAM50 algorithm (Parker et al., 2009). PAM50 classification of this dataset was achieved previously in de Anda-Jáuregui et al. (2015, 2016).

### Network inference

Gene regulatory network inference from experimental data has been extensively used to unveil interactions between genes from their experimentally-measured expression levels. ARACNE (Margolin et al., 2006) is one of the most employed algorithms to calculate correlations between pairs of genes. In a nutshell, the algorithm calculates the *Mutual Information (MI)*—a non-parametric measure that captures non-linear dependencies between variables (Hernández-Lemus and Siqueiros-Garćıa, 2013)—in a relatively fast implementation. The method associates a *MI* value to each significance value (*p*-value) based on permutation analysis, as a function of the sample size.

In our case, we described previously the network architecture of the 4 breast cancer molecular subtypes (de Anda-Jáuregui et al., 2016). In that paper, (1) it was calculated the MI for every pair of (non-self) probe sets in the microarray platform; (2) a MI cutoff proportional to p-value, corrected for sample size was established; (3) Probe sets were mapped to HUGO gene symbols, discarding those without a corresponding gene symbol.

We also performed other three methods for network inference, based on linear correlations, namely, Pearson, Spearman and Kendall correlation measures. Additionally, we inferred mutual information-based networks by using CLR and MRNETB algorithms (Supplementary Material 1). All of these were executed with the R package minet (Meyer et al., 2008).

### Module detection

There are many methods and techniques for community detection (Girvan and Newman, 2002; Clauset et al., 2004; Palla et al., 2005; Newman, 2006; Rosvall and Bergstrom, 2007; Fortunato, 2010; Fortunato and Hric, 2016), we decided to use *Infomap* (Rosvall and Bergstrom, 2008) since it has shown to be one of the best suited algorithms for detecting modules both in performance and accuracy (Lancichinetti et al., 2009) as it was assessed in terms of the *LFR benchmark* (Lancichinetti et al., 2008).

In the same way in which countries, regions or cities correspond to structures with more information on a map than, say a single street; Infomap identifies the relevant structures in the network as if making a map of it from the flow of information between these structures. This is achieved by compressing the information described by a random walk inside the network, being that a random walker will spend more time in a structure with greater internal information flow in the network (a greater density of internal edges) before jumping to another, as happens when navigating between cities or countries.

The description of this walk is made using Huffman coding (Huffman et al., 1952), thus it is possible to recover the most important structures of the network by minimizing this description using information theory; just like cities that have a shorter description on a map contain much more information than a particular address whose length is longer. Hence, we applied Infomap to each of the connected components of each network, previously inferred in de Anda-Jáuregui et al. (2016), for the four breast cancer molecular subtypes—since the detection of modules only makes sense in connected networks ^1^—with the aim to glimpse the modular structure of them.

Additionally, to corroborate that the network topology as well as the modular structure found with our approach are due to the intrinsic nature of tumor biology, we constructed null models for each network. In this null model, all nodes and edges for each molecular subtype network were maintained, but randomly rewired, according to the Erdös-Rényi model (Erdös and Rényi, 1959). Additionally, we constructed another null model following the method previously published in Faith et al. (2007) based on Newman et al. (2001) and Newman (2003), to preserve the same degree distribution than our inferred networks (Supplementary Material 2).

### Gene ontology enrichment

The basic hypothesis in an overrepresentation analysis (ORA), is that relevant pathways can be detected if the proportion of differentially expressed genes, within a given pathway, exceeds the proportion of genes that could be randomly expected (Garćıa-Campos et al., 2015). We performed ORA for each module, by resorting to FDR-corrected hypergeometric tests with HTSanalyzeR (Wang et al., 2011), choosing a significance below Q-value = 0.001. Categories larger than 1000 genes were discarded.

### Functional analysis

Modules were labeled according to the highest PageRank gene, i.e., each module is named by its top-PageRank centrality gene. For each subtype, we observed the number of modules and their respective size. We also observed whether or not modules are conserved across molecular subtypes to assess the existence of common regulatory process in breast cancer transcriptional programs. The hypothesis is that different network architectures determine specific community structures and hence, different processes would control the regulatory program for each molecular subtype.

We looked up for general GO categories (i.e., main branches in the ontology tree) representative of the basal subtype regulatory network. We focused on this subtype since it does not present therapeutic alternatives but cytotoxic or surgery. By identifying the processes enriched for their communities, we analyzed how specific those processes are. On the other hand, we also observed whether a process/processes appear in several modules or subtypes, providing insights regarding concomitant processes that occur in cancer, despite the network architecture (as given by the molecular subtype).

Once the functional analysis were performed we studied those genes with highest centrality measures: *Betweenness centrality, clustering coefficient, shortest path length, degree and PageRank*, to evaluate with more accuracy the role of those genes in the module and more importantly, in the biological process.

Finally, taking into account the differentially expressed genes, we investigated whether these expression patterns could activate or inhibit the statistically significant enriched processes. By means of z-score calculations (Krämer et al., 2014) we assessed the degree of dysregulation of said processes.

## Results and discussion

### Modular structure is specific for each breast cancer subtype network

By means of mutual information-based network inference for the 4 breast cancer subtypes, we previously described (de Anda-Jáuregui et al., 2016) how differences in the transcriptional program between breast cancer molecular subtypes, reflected in different transcriptional network architectures. Table 1 briefly summarizes some of these structural differences.

**Table 1:** Network parameters for the modular structure of breast cancer molecular subtypes.

**Subtype**	**Number of components with enriched modules**	**Number of enriched modules**	**Number of nodes in the largest component**	**Number links in the largest component**
Luminal A	6	8	930	8,535
Luminal B	3	5	555	8,476
Basal	4	7	523	7,181
HER2-enriched	1	3	1,649	9,108

*Number of components with enriched modules, enriched modules, number of nodes in the largest connected component, as well as links in the largest connected component are shown*.

From this table, it is possible to observe differences between the architectures for each network subtype. Considering these different topologies, we observe that each transcriptional network presents a characteristic modularity pattern, i.e., the gene composition of communities and the connectivity rules for each subtype are unique.

**Figure 2:**
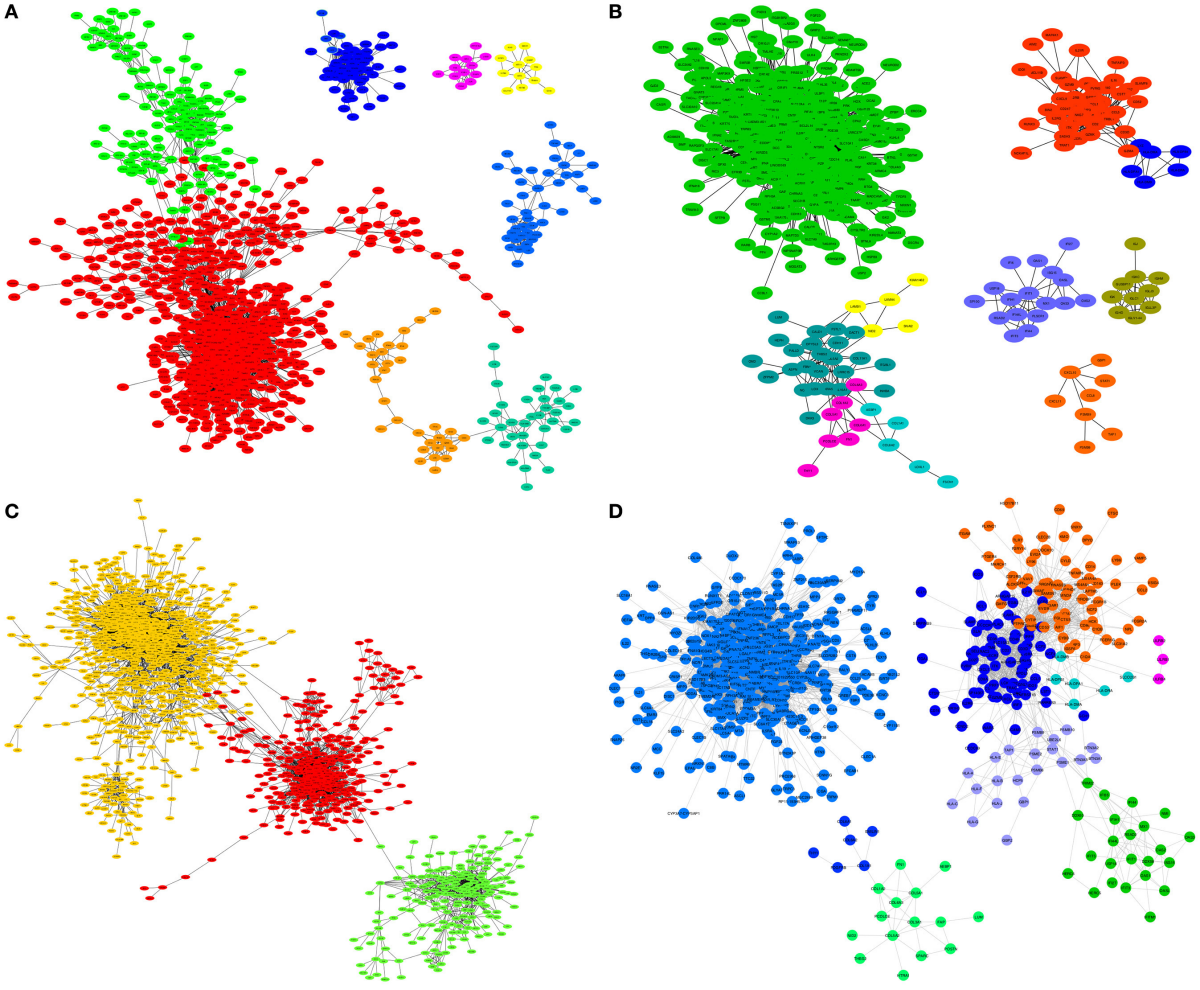
Modules in networks for each breast cancer molecular subtype. **(A)** Luminal A; **(B)** Luminal B; **(C)** HER2+ and **(D)** Basal subtype. The nodes belonging to a community are colored the same. The same color for different subtypes is not related. For visualization purposes, only islands with more than 8 genes are depicted.

Table 1 also shows that the number of modules for each subtype is different. In Figure 2, we show a visualization of each transcriptional network, with nodes colored by module. A remaining question is whether or not those modules have a specific phenotype-dependent functional role in the regulatory program of each breast cancer molecular subtype.

With the module detection method used in this work, all network's genes are classified as an element of a module. For this reason, we have modules with associated Gene Ontology processes, but also modules which do not have statistically significant enriched categories.

Of course, different sizes of networks will have different topological parameters. In the case of mutual-information inference of networks, it is necessary to establish a threshold for valid interactions. To have large networks, a low cutoff value is needed; on the contrary, for small networks, it is mandatory to have a strict threshold of mutual information. In de Anda-Jáuregui et al. (2016) it was demonstrated that the global properties of the four networks for the breast cancer molecular subtypes are conserved throughout a large range of network sizes (3 orders of magnitude). For this reason, we performed the module detection algorithm as well as the functional enrichment with the same networks than those published in de Anda-Jáuregui et al. (2016) (top-10,000 links).

### GO categories are associated to modules for each molecular subtype

In Table 2, it can be observed how each molecular subtype has a unique set of enriched modules, regarding module sizes and number of modules, as well as the number of enriched processes for each community. It is worth mentioning that the number of enriched processes is not directly related to the size of the modules (From now on, we will name the modules according to its top-PageRank node with the nomenclature Name_of_the_Gene_*comm*_).

**Table 2:** Enriched modules found in transcriptional networks for breast cancer molecular subtypes.

	**Community**	**Number of genes**	**Number of enriched processes**
	LUZP4	805	8
	NFIC	125	20
	**COL5A2**	**53**	**21**
Luminal A	CD2	33	8
	TYROBP	36	3
	PLIN1	42	1
	KRT14	12	4
	ZFP36	12	12
	LUZP4	464	15
	CD2	42	4
Luminal B	**COL5A2**	**24**	**6**
	IFIT1	17	7
	IGKC	10	6
	CNR2	846	6
HER2+	LCK	370	83
	**COL5A2**	**196**	**29**
	SLC4A4	390	7
	CD2	72	15
	CD53	65	3
Basal	RSAD2	23	9
	PSMB9	21	38
	**COL5A2**	**15**	**16**
	IGLC1	12	6

*Modules are labeled according to their top-PageRank genes. Number of genes as well as number of enriched processes per community are shown. Notice that the four subtypes contain a module in which COL5A2 gene is the top-PageRank node (bold), although those modules are different*.

For instance, LUZP4_*comm*_ for the luminal A subtype is composed by 805 genes, however, only 8 processes are enriched for this module. On the other hand, ZFP36_*comm*_ contains only 12 genes, but 12 processes are enriched on it. Processes enriched for ZFP36_*comm*_ are related to the response to stimulus and signaling.

Interestingly, each module in the luminal A network has a unique set of enriched processes. This could be related to the network structure (Figure 2A), since almost all modules are separated from each other. This is not the case for the rest of molecular subtypes. The full list of enriched processes per module for all molecular subtype networks can be found in Supplementary Material 3.

#### COL5A2_*comm*_ is present in each molecular subtype

We mentioned previously that the community structure for each molecular subtype network is different. And this could be related to the aforementioned specific behavior observed in each phenotype. Notwithstanding, breast cancer have a common core of features that can be mapped to the genetic regulatory program (Espinal-Enríquez et al., 2017a), commonly named as *hallmarks of cancer* (Hanahan and Weinberg, 2011).

**Figure 3:**
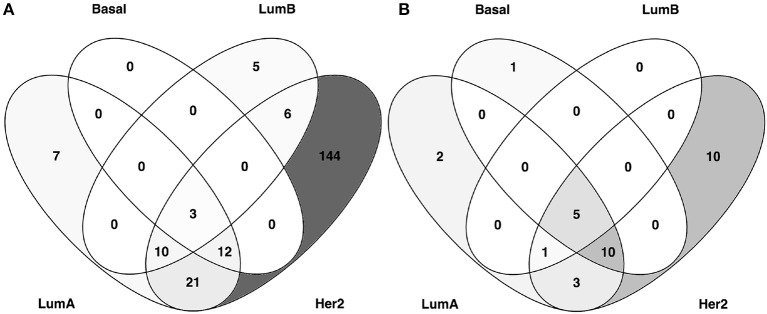
COL5A2 communities: Enriched processes are shared between subtypes despite gene compositions being different. **(A)** Venn diagram showing the number of genes of COL5A2_*comm*_ for each molecular subtype. Notice that only 3 genes (*COL5A2, THBS2*, and *LUM*) are shared. **(B)** Enriched processes of COL5A2_*comm*_ for each molecular subtype.

In the particular case of these inferred networks, we want to stress the case of the “COL5A2 communities” (bold in Table 2). In each subtype, we identified a module in which the most PageRank gene was *COL5A2*, the gene for the collagenase 5a2 protein, an integral component of the extracellular matrix (ECM). However, the gene composition for COL5A2_*comm*_ is different among molecular subtypes. It can be observed in Figure 3A, where a Venn diagram of the gene composition of COL5A2_*comm*_ for each molecular subtype is shown.

A series of features of this diagram can be discussed: each module contains a different number of genes, but more important, there are only three genes that are shared between all subtype networks: *COL5A2, THBS2*, and *LUM*. *THBS2* codifies to the thrombospondin-2 a well-known cell-cell communicator and inhibitor of tumor growth and angiogenesis, which has been associated with both gastric and breast cancer (Koch et al., 2011; Sun et al., 2014). *LUM* gene, codifies to lumican stromal protein, which in turn regulates collagen fibril organization; genomic variations of this gene have been associated with breast cancer (Kelemen et al., 2008).

Nevertheless, in each subtype, the COL5A2_*comm*_ is associated with similar processes, as shown in of Figure 3B. There are five enriched processes common to COL5A2_*comm*_ across subtypes: collagen fibril organization, extracellular matrix (ECM), extracellular matrix organization, extracellular matrix structural constituent, and extracellular region. This may imply that ECM-related processes are a common feature of breast cancer, via *COL5A2* gene regulatory program, and this is independent of the molecular subtype (a table containing the gene lists of COL5A2 communities can be found in Supplementary Material 4).

By looking at the *COL5A2*-associated modular structure in the different subtypes, we were able to discern the existence of a specific set of processes common to all breast cancer subtypes: ECM dysregulation. The appearance of common enriched processes is independent of the genes present in each subtype network. This is a clear instance of the robustness of crucial processes acquired during breast cancer development; the relevance of ECM dysregulation may exert on cancer malignancy and progression is well known (Bonnans et al., 2014; Espinal-Enríquez et al., 2015a).

Also, given the strong relationship of ECM with several other processes during cancer development, such as angiogenesis (found in HER2+ COL5A2_*comm*_), immunity or cell migration (reviewed in Bonnans et al., 2014), this finding acquires more relevance. COL5A2 modules have ECM-related processes enriched by different genes. Those genes also contribute to the enrichment of other processes which may shape specific landscapes across molecular subtypes.

For instance, HER2+ subtype COL5A2_*comm*_ has 10 unique significantly-enriched processes, including TGF-β signaling, focal adhesion, or angiogenesis. Enrichment occurs via genes such as TGF-β, metalloproteinases, collagenases, VCAN or fibronectin which, appear in the COL5A2_*comm*_ of the HER2+ subtype network.

TGF-β in particular, is quite relevant to favor and promote a metastatic environment (Ghajar et al., 2013). TGF-β is also directly active in the SMAD translocation into nucleus, promoting the expression of several ECM-related molecules (Verrecchia et al., 2001). Despite the differences between genes for each COL5A2_*comm*_ per subtype, ECM-related processes appear consistently, and the non-common processes for each subtype may reflect particular environments involved in the tumor biology for each subtype.

### COL5A2 modules are consistently overexpressed across all subtypes

**Figure 4:**
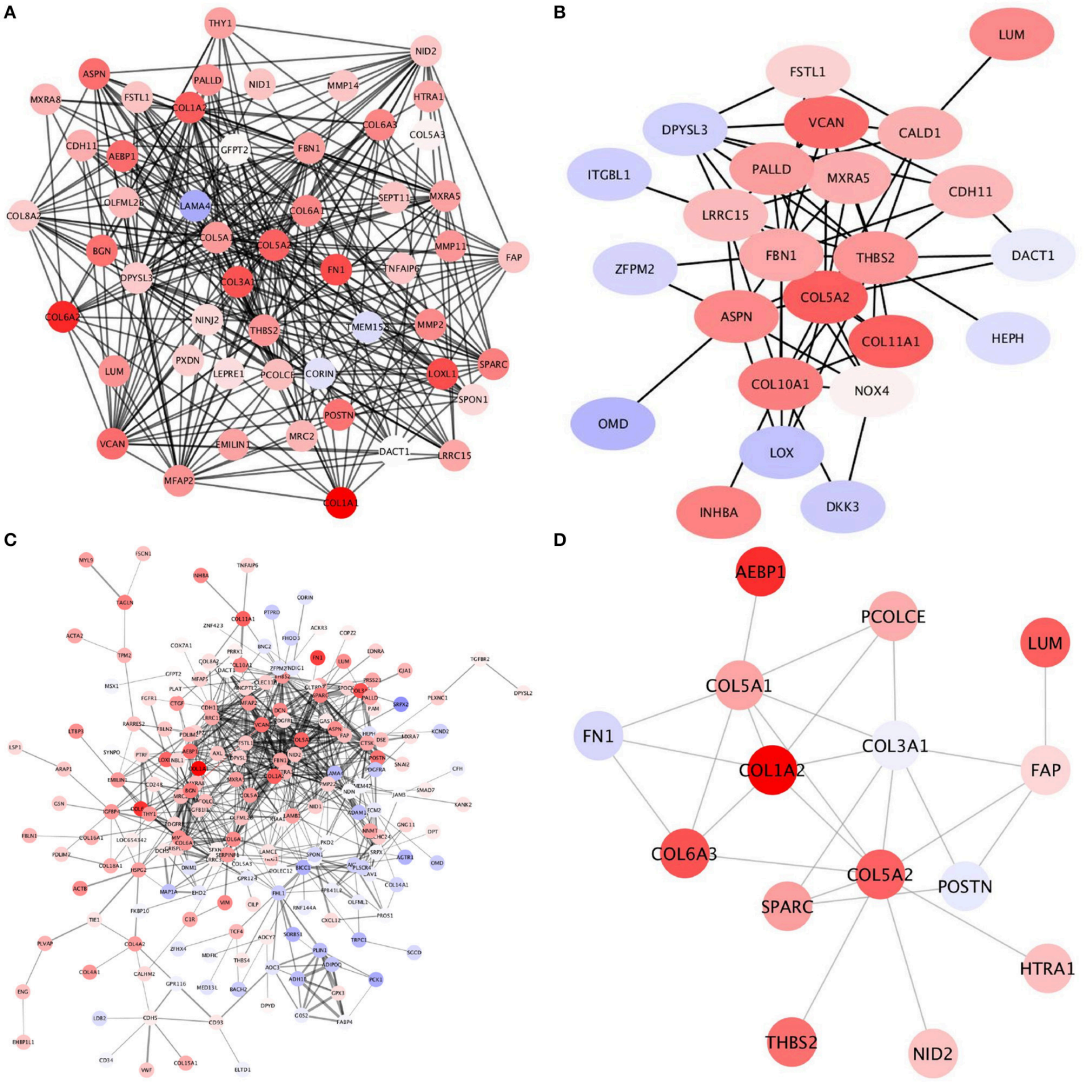
Genes in COL5A2 modules are mostly overexpressed thruoghout molecular subtypes This figure shows the expression signature of those genes belonging to COL5A2 modules in **(A)** Luminal A **(B)**, Luminal B **(C)**, HER2+, and **(D)** Basal breast cancer molecular subtypes. Notice that the majority of genes are overexpressed. However, in **(C)** there is a subset of genes which is underexpressed and are grouped in terms of the network topology.

Acknowledging that the expression pattern is crucial to infer the effect of a gene regulatory network, we explored the expression profile of the COL5A2 modules in each subtype. Interestingly, for all subtypes, despite those modules contain different genes, all subtypes have an overexpression pattern, this is clearly observed in Figure 4. There, genes are colored according to their expression levels (red for overexpressed and blue for underexpressed).

As it can be observed, the COL5A2 module in each subtype reflects that those enriched processes are exacerbated, which is consistent with the fact that ECM-related processes are up-regulated, corroborating the previous observations.

### Basal subtype modules analysis

We have now explored some of the differences in the regulatory programs behind common breast cancer subtypes, as seen in their underlying transcriptional networks. Based on the association between the modular structures in these networks and biological processes, we have shown that each molecular subtype has a specific functional landscape, which may be associated with the features observed in the clinical setting.

**Figure 5:**
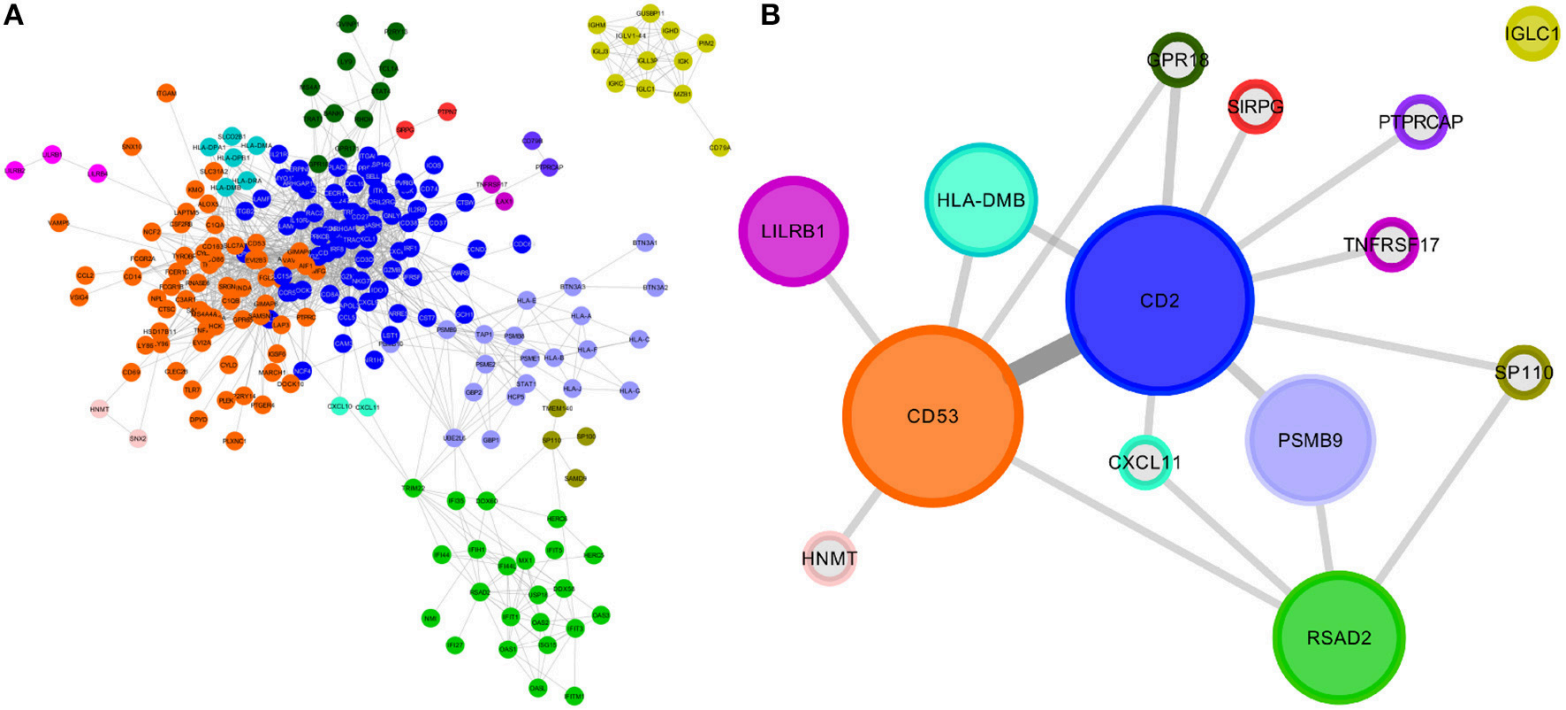
Modular structure of Basal subtype islands 2 and 6. **(A)** Colors define each module. **(B)** Information flow between communities. Link width is proportional to the number of links shared between modules. Full-color communities represent those that are enriched to a GO category.

With this in mind, we will now focus on the study of the basal molecular subtype. As described in the introduction, this is the most malignant subtype of breast cancer, with the poorest prognosis, and the most restrictive therapeutic alternatives. In Figure 5A we can observe the module structure of two islands of basal subtype. Colors represent each module. We show the second largest component and the IGLC1_*comm*_, that is not connected to the larger island, but it shares enriched processes with that island.

**Figure 6:**
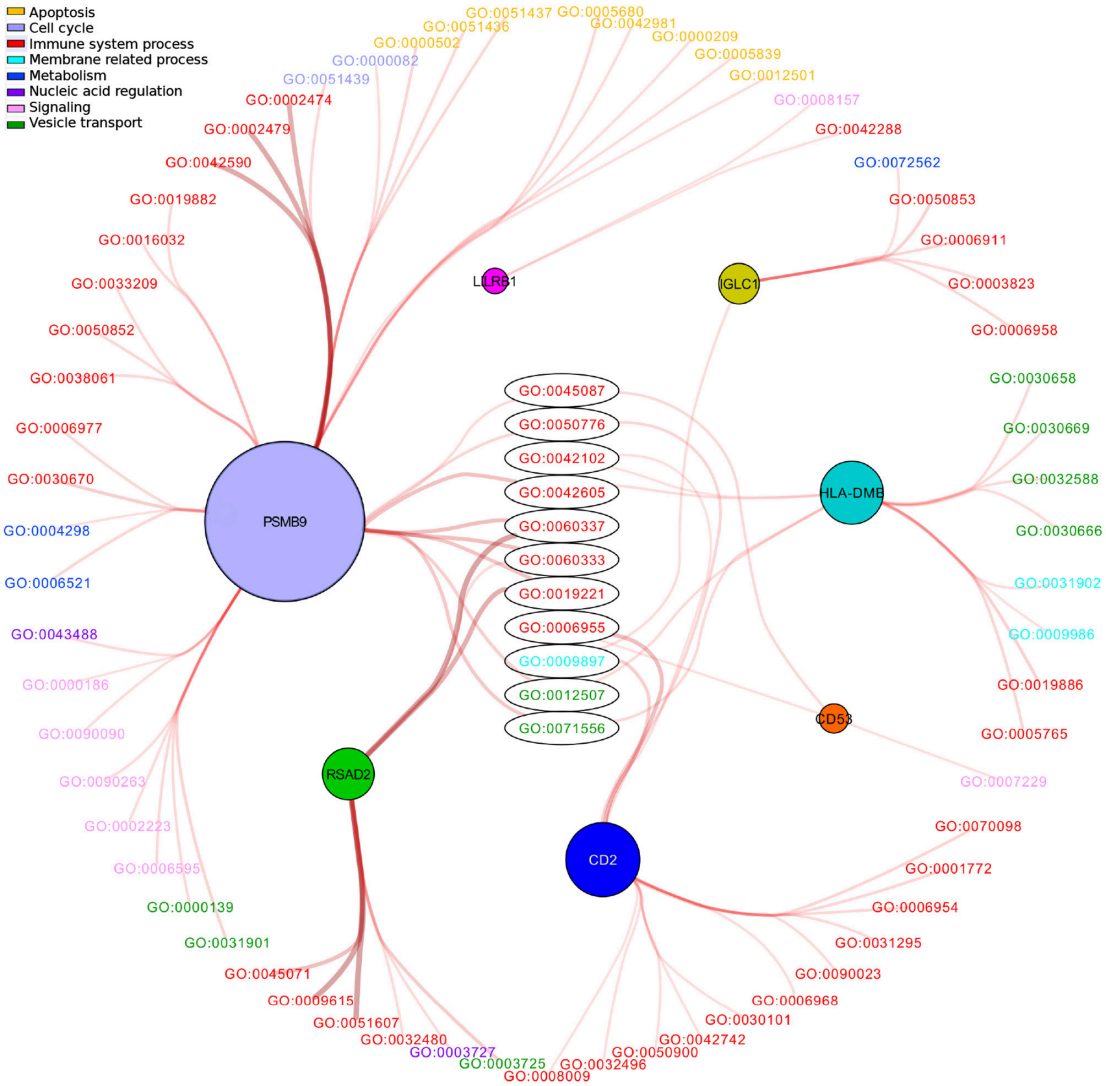
GO processes associated with modules in the transcriptional network of basal breast cancer molecular subtype. In this figure, modules are colored according to the color code of Figure 5. These communities are connected to GO ID categories which are colored according to a general process (upper left). Names of those categories are provided in Supplementary Material 5.

Figure 5B shows the enriched modules (color-filled nodes). Regarding the enriched modules found in Figure 5, we observed which GO categories are involved for each module. The results are depicted in Figure 6. There, modules are colored according to the color code of Figure 5, meanwhile labels of enriched processes are colored depending on the general type of the process.

From a visual inspection of Figure 6, it is clear that the majority of enriched categories of those modules are related to immune system processes (red labels). However, some modules have other enriched processes, such is the case of PSMB9_*comm*_, which contains only 21 genes, but 38 GO categories are enriched by it. This is the only module in which apoptosis-related enriched processes (yellow labels) can be observed. Regarding enriched categories of PSMB9_*comm*_ we also observe:

- 8 out of the 38 PSMB-enriched processes are related to apoptosis. There is no other module which contains enriched apoptosis-related processes: yellow names in Figure 6 are only connected to PSMB9_*comm*_.
- PSMB9 also has another 29 unique processes related to it.
- 5 signaling processes are also unique to PSMB9_*comm*_.
- Apoptosis-related categories are involved in the proteasome complex and its regulation. The whole list of processes is shown in Supplementary Material 5.

It is also worth mentioning that 40 out of 74 enriched GO categories belong to immune system-related processes; 32 of them are unique categories for independent modules. This may imply that despite modules being sets of nodes more connected between them over the rest of the component, these communities indeed communicate with each other, and present a complete regulatory program in which specific modules act individually as a part of a whole, at least, regarding immunity. The 11 shared processes (8 of them related to immunity) at the center of the figure reinforce this suggestion.

### PSMB9_*comm*_ and decreasing of apoptosis

The network modular structure found here, reflects not only how groups of genes participate in an orchestrated functional process, but also how the expression patterns of those genes are according to the specific direction in which said process functions, namely, increasing or decreasing. As an example of this last, we mention the case of PSMB9_*comm*_ in basal subtype.

As we previously mentioned, PSMB9_*comm*_ is composed by 21 genes, but that module is enriched in 38 processes, mostly related to apoptosis and immunity (Supplementary Material 7). Furthermore, network centralities in this module reveal the relevance of some genes in terms of flux of information and a coordinated regulation of sub-processes. For example, PSMB9, TAP1 and UBE2L6 are the genes with highest *Betweenness centrality, clustering coefficient, degree* and *PageRank* centralities. Because of these properties, removal of them divide the subnetwork in two modules: on the one hand, the strongly-connected module of HLA genes, mainly related to the *Major Histo-Compatibility Complex (MHC)*, and on the other hand, the *proteasome complex*, mainly related to apoptosis and ubiquitination. Based on several centrality measurements, we can argue that these elements are regulating in a coordinated fashion both processes, immunity and cell death (see Supplementary Material 6).

Also important is the fact that all genes in the module are overexpressed (Supplementary Material 5). Having in mind that cell death and immunity act coordinately in basal subtype, we investigated the predicted activation or inhibition that those processes present, i.e., based on the expression profile of the geneset, which is the direction of change for a determined function. In this case, Cell death and immunity. For this purpose, we used the Diseases and Functions Analysis provided by QIAGEN's Ingenuity® Pathway Analysis (IPA®, QIAGEN Redwood City, www.qiagen.com/ingenuity), which assesses (by means of a z-score) the match between observed and predicted up/down regulation patterns, as described in Krämer et al. (2014) and Espinal-Enríquez et al. (2015a).

**Figure 7:**
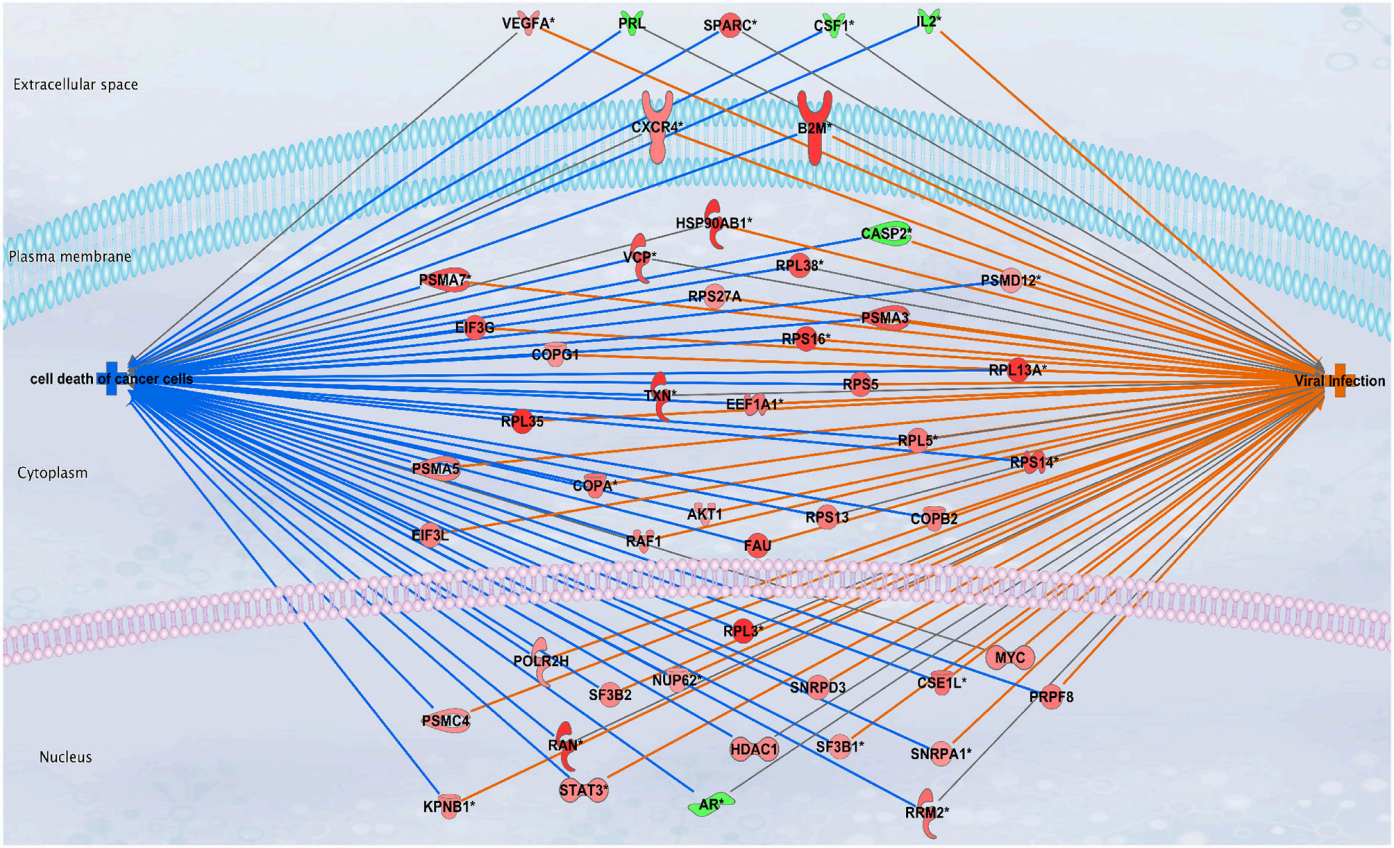
Cell death and viral infection processes are oppositely regulated by the same molecular signature of basal breast cancer subtype. In this figure, genes are depicted according to their expression levels: red for overexpressed and blue for underexpressed genes. Lines between molecules and processes indicate the predicted function of the molecule according to its expression value, blue line leads an inhibition of the process; in turn, orange lines account for predicted activation. Color of cell death and viral infection processes represent the same predicted effect than lines.

Interestingly, the most inhibited function predicted by the analysis is Cell death (z-scores of -8), meanwhile the most activated function, predicted by the analysis (z-scores of 7) was viral infection. This means that the expression profile of molecules related to both processes has an expression signature observed when cell death-related events are decreased and concomitantly, genes responding to a viral infection activate a defense scheme. Figure 7 shows a subset of molecules that participate in both processes and have a predicted effect on them. As it can be observed, the same expression signature, inhibits cell death and at the same time, it seems to activate viral infection genes.

## Concluding remarks

Here, by adopting a Systems Biology approach, we inferred transcriptional networks for breast cancer molecular subtypes, we find the modular structure for each network, and performed enrichment analyses for all the detected modules. All information regarding networks as well as modularity performed in this work can be found in Supplementary Material 8: the .cys file of all networks. As a summary, we present the most relevant results obtained with our approach:

- Modularity of each breast cancer molecular subtype network is different.
- There is a unique pattern of enriched processes associated with modules for each subtype network.
- Despite these particular community structures, COL5A2_*comm*_ is present in all subtypes, furthermore, even if those modules do not have the same genes, they share processes related to extracellular matrix and collagen fibril formation, suggesting robustness in processes for breast cancer in general, independent of the participating genes.
- For basal subtype modular structure, there are several unique processes which are related to apoptosis and immune system.

Inference of transcriptional networks using high throughput experiments is still an open problem. We are aware that many algorithms exist, which may generate different results since they are based on different assumptions (for further review of this topic, refer to Hernández-Lemus and Siqueiros-Garćıa, 2013). In our previous work (de Anda-Jáuregui et al., 2016) we used ARACNE, which resulted suitable to generate comparable network models for breast cancer molecular subtypes. The discussion in the present work was built upon these previous results. In Supplementary Material 1, we provide networks inferred by using other algorithms for basal subtype, for comparison purposes.

As it can be observed from the analysis, the structure of the gene regulatory networks for breast cancer subtypes is strongly associated to function. This association may not be directly observed, unless analyzed by an approach such as the provided here. Regarding the basal subtype the tight co-regulation of genes participating together in apoptosis-related processes may open a possibility to explore therapeutics targeting those molecules or downstream elements of them.

Immunity in basal subtype has not been profoundly explored. A methodology such as the one presented here, can be combined with different analyses in order to integrate multiple sources of information into a solid framework. An example of this can be found in (Li et al., 2015), where Genome-Wide Association Studies (GWAS) were combined with CD4+ master regulator genes, to identify possible mechanisms of appearance of aberrant phenotypes.

We were able to reduce the degrees-of-freedom of our data. From hundreds of experiments of microarrays (493), from inferred transcriptional regulatory networks (de Anda-Jáuregui et al., 2016), which identified the top 10,000 interactions between genes (from 220,000,000 of possible interactions). Using that information, we identified modular structures, related to the manner in which genes are interconnected. That architecture is not only relevant for topological analysis, but also for biological functionality. Further work includes possible applications into different datasets, RNA-seq technology, other cancers and possible experimental procedures to investigate the implication of apoptosis-related processes and immunity in the breast cancer basal subtype.

## Author contributions

SAA-C, GdA-J, and JE-E devised the experiment, performed the calculations, analyzed the results, contributed to the discussion, designed the figures and wrote the manuscript. EH-L proposed the experiment, participated in its design and coordination, contributed to the discussion, supervised and reviewed the writing of the manuscript. All authors read and approved the final manuscript.

### Conflict of interest statement

The authors declare that the research was conducted in the absence of any commercial or financial relationships that could be construed as a potential conflict of interest.

## Footnotes

^1^A network divided into components has been indeed already partitioned.
