# Supplementary figures and images for: Network Modularity in Breast Cancer Molecular Subtypes
Source: Front Physiol. 2017 Nov 17;8:915. doi: 10.3389/fphys.2017.00915 (PMC5699328; doi:10.3389/fphys.2017.00915)

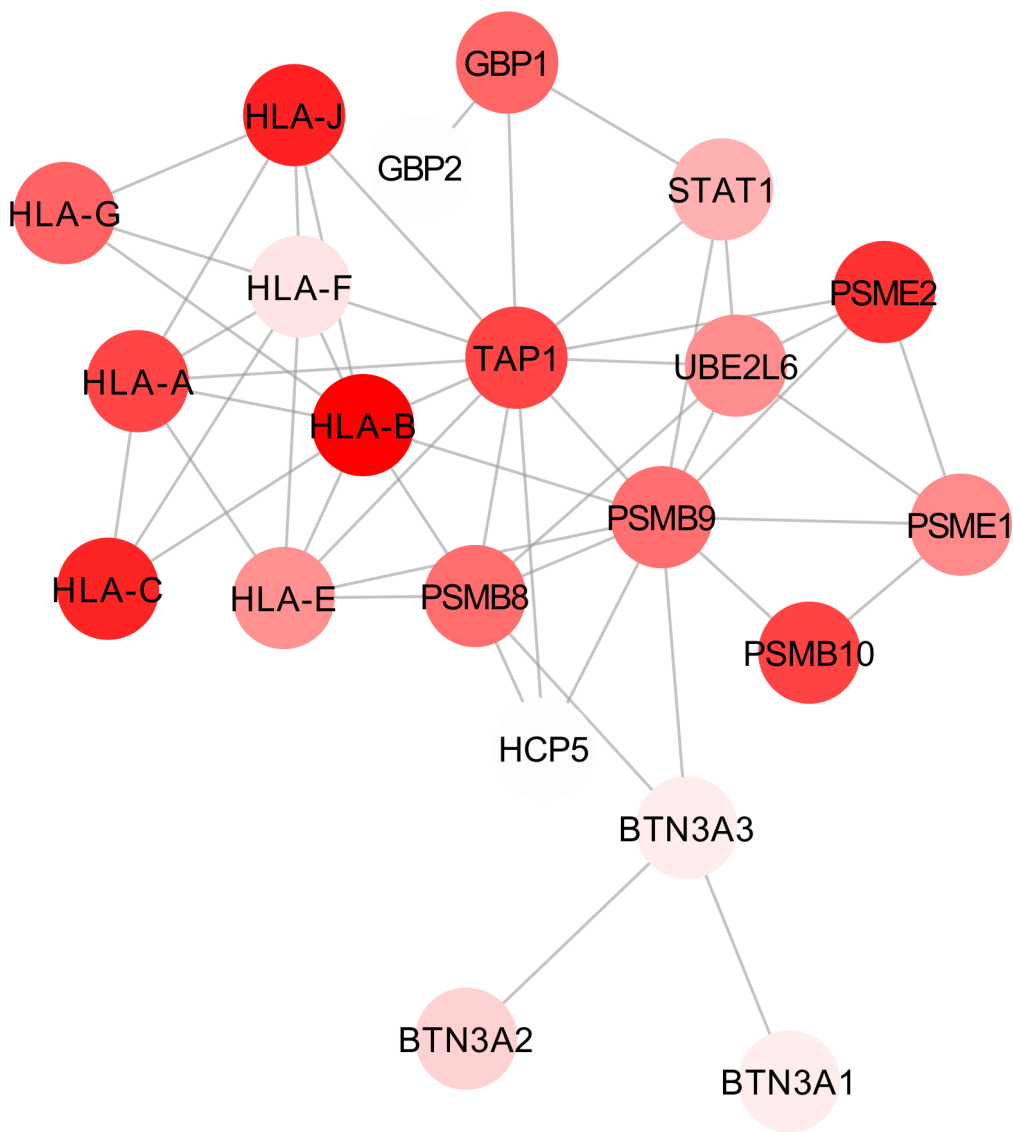

Supplement: Supplementary file 6 [file SupplementaryMaterial6.PDF]
