# Supplementary material for: Network Modularity in Breast Cancer Molecular Subtypes
Source: Front Physiol. 2017 Nov 17;8:915. doi: 10.3389/fphys.2017.00915 (PMC5699328; doi:10.3389/fphys.2017.00915)
Supplement: Supplementary file 7 [file SupplementaryMaterial7.PDF]

Top nodes by centrality in relevant modules

COL5A2

**Degree / PageRank**							
**HER2**		**LumA**		**LumB**		**Basal**	
FBN1	38	COL5A2	36	COL5A2	22	COL5A2	10
COL5A2	37	COL5A1	30	THBS2	17	COL3A1	7
VCAN	33	FBN1	29	FBN1	17	COL5A1	7
THBS2	32	THBS2	29	LRRC15	12	COL1A2	7
COL1A2	32	DPYSL3	28	VCAN	10	COL6A3	4
**Clustering Coefficient**							
**HER2**		**LumA**		**LumB**		**Basal**	
DNM1	1	GFPT2	1	LOX	1	PCOLCE	1
PALLD	1	BGN	1	COL11A1	1	FN1	1
COL8A2	1	POSTN	1	ZFPM2	1	COL6A3	0.83333333
MXRA7	1	SEPT11	1	DKK3	1	SPARC	0.66666667
PRSS23	1	HTRA1	1	PALLD	0.9	POSTN	0.66666667
**Betweenness**							
**HER2**		**LumA**		**LumB**		**Basal**	
HSPG2	0.16262	THBS2	0.12928155	COL5A2	0.34761256	COL5A2	0.49307359
BGN	0.06693	MFAP2	0.11665086	FBN1	0.14697318	COL1A2	0.44906205
BICC1	0.06528	COL5A2	0.111609	THBS2	0.11467705	COL3A1	0.14704185
DPYD	0.05083	FBN1	0.1040877	LRRC15	0.09701108	COL5A1	0.12438672
ADCY7	0.05081	FN1	0.08563821	COL10A1	0.08191115	FAP	0.1004329
**<SPL>**							
**HER2**		**LumA**		**LumB**		**Basal**	
DPYSL2	12.4387	NID1	3.62365591	LUM	3.5	LUM	3.5
MYL9	12.0036	COL5A3	3.41935484	OMD	3.44642857	AEBP1	3.04545455
FSCN1	12.0036	MMP14	3.41935484	INHBA	3.35714286	POSTN	2.86363636
TGFBR2	11.4393	TMEM158	3.34408602	DKK3	3.30357143	NID2	2.81818182
ACTA2	11.0067	MMP11	3.34408602	ITGBL1	3.17857143	HTRA1	2.81818182
**Closeness (1/<SPL>)**							
**HER2**		**LumA**		**LumB**		**Basal**	
HSPG2	0.1545	COL5A2	0.53142857	COL5A2	0.53333333	COL5A2	0.53658537
LRRC32	0.14408	FBN1	0.51666667	THBS2	0.47457627	COL1A2	0.53658537
BGN	0.14035	MFAP2	0.51098901	FBN1	0.46280992	COL3A1	0.48888889
MMP2	0.13984	DPYSL3	0.5	LRRC15	0.45528455	COL5A1	0.47826087
PDGFRB	0.13979	COL5A1	0.4973262	MXRA5	0.448	COL6A3	0.44
**Eccentricity**							
**HER2**		**LumA**		**LumB**		**Basal**	
DPYSL2	19	COL6A3	6	LUM	7	LUM	6
MYL9	19	MMP2	6	CDH11	6	FAP	5
FSCN1	19	LEPRE1	6	CALD1	6	SPARC	5
TGFBR2	18	TNFAIP6	6	PALLD	6	NID2	5
ACTA2	18	BGN	6	LOX	6	HTRA1	5

PSMB9

**Degree / PageRank**	
**Basal**	
PSMB9	19
TAP1	17
UBE2L6	14
HLA-B	9
PSMB8	8
**Clustering Coefficient**	
**Basal**	
HCP5	1
HLA-C	1
HLA-G	1
HLA-J	0.8
HLA-A	0.73333333
**Betweenness**	
**Basal**	
PSMB9	0.10975816
UBE2L6	0.05758558
TAP1	0.04406813
BTN3A3	0.01864771
HLA-B	0.01647491
**<SPL>**	
**Basal**	
GBP2	4.78504673
BTN3A1	4.51869159
BTN3A2	4.51869159
HLA-C	4.45327103
HLA-G	4.45327103
**Closeness (1/<SPL>)**	
**Basal**	
PSMB9	0.39194139
UBE2L6	0.36896552
TAP1	0.3434992
PSMB10	0.3354232
STAT1	0.32522796
**Eccentricity**	
**Basal**	
GBP2	8
BTN3A1	8
BTN3A2	8
HLA-C	8
HLA-G	8

LUZP4

**Degree / PageRank**			
**LumA**		**LumB**	
LUZP4	235	LUZP4	242
CHODL	195	DCT	225
UBE2D4	186	GPRC5D	211
CYP2C9	176	KRT2	192
PSG9	173	GPA33	190
**Clustering Coefficient**			
**LumA**		**LumB**	
BMP6	1	OR7E12P	1
TYR	1	FMO4	1
CBLN1	1	CPA2	1
MIA2	1	KRT9	1
ODF1	1	MYOZ2	1
**Betweenness**			
**LumA**		**LumB**	
DMWD	0.10451977	LUZP4	0.07433887
LUZP4	0.15417349	GPRC5D	0.05675599
CHODL	0.20385937	DCT	0.0554385
FLRT1	0.54347826	ZNF507	0.04141004
UBE2D4	0.22441151	MAP3K19	0.04049259
**<SPL>**			
**LumA**		**LumB**	
CCNL2	10.63832078	DSCR4	3.91696751
SUGP2	9.6393972	USP2	3.66064982
SPG7	8.64262648	SSTR4	3.62635379
CNOT8	7.67599569	NRXN1	3.51624549
TMEM50B	7.67599569	CSN3	3.51263538
**Closeness (1/<SPL>)**			
**LumA**		**LumB**	
LUZP4	0.40853122	LUZP4	0.58132214
CHODL	0.40532286	DCT	0.57172343
UBE2D4	0.39633106	GPRC5D	0.56415479
CCDC7	0.39165261	KRT2	0.5352657
SCN10A	0.37672344	GPA33	0.53116012
**Eccentricity**			
**LumA**		**LumB**	
CCNL2	15	PRO2012	11
GPR171	14	NRXN1	11
SIRPG	14	USP2	11
MRPS28	14	DSCR4	11
CNOT8	14	NTNG1	10
